# Effects of repetitive transcranial magnetic stimulation and trans-spinal direct current stimulation associated with treadmill exercise in spinal cord and cortical excitability of healthy subjects: A triple-blind, randomized and sham-controlled study

**DOI:** 10.1371/journal.pone.0195276

**Published:** 2018-03-29

**Authors:** Plínio Luna Albuquerque, Mayara Campêlo, Thyciane Mendonça, Luís Augusto Mendes Fontes, Rodrigo de Mattos Brito, Katia Monte-Silva

**Affiliations:** 1 Applied Neuroscience Laboratory, Department of Physical Therapy, Universidade Federal de Pernambuco, Recife, Pernambuco, Brazil; 2 Department of Physical Therapy, Centro Universitário Tabosa de Almeida, Caruaru, Pernambuco, Brazil; 3 Postgraduate Program in Neuropsychiatry and Behavioral Sciences, Universidade Federal de Pernambuco, Recife, Pernambuco, Brazil; Shanghai Mental Health Center, CHINA

## Abstract

Repetitive transcranial magnetic stimulation (rTMS) over motor cortex and trans-spinal direct current stimulation (tsDCS) modulate corticospinal circuits in healthy and injured subjects. However, their associated effects with physical exercise is still not defined. This study aimed to investigate the effect of three different settings of rTMS and tsDCS combined with treadmill exercise on spinal cord and cortical excitability of healthy subjects. We performed a triple blind, randomized, sham-controlled crossover study with 12 healthy volunteers who underwent single sessions of rTMS (1Hz, 20Hz and Sham) and tsDCS (anodal, cathodal and Sham) associated with 20 minutes of treadmill walking. Cortical excitability was assessed by motor evoked potential (MEP) and spinal cord excitability by the Hoffmann reflex (Hr), nociceptive flexion reflex (NFR) and homosynaptic depression (HD). All measures were assessed before, immediately, 30 and 60 minutes after the experimental procedures. Our results demonstrated that anodal tsDCS/treadmill exercise reduced MEP’s amplitude and NFR’s area compared to sham condition, conversely, cathodal tsDCS/treadmill exercise increased NFR’s area. High-frequency rTMS increased MEP’s amplitude and NFR’s area compared to sham condition. Anodal tsDCS/treadmill exercise and 20Hz rTMS/treadmill exercise reduced Hr amplitude up to 30 minutes after stimulation offset and no changes were observed in HD measures. We demonstrated that tsDCS and rTMS combined with treadmill exercise modulated cortical and spinal cord excitability through different mechanisms. tsDCS modulated spinal reflexes in a polarity-dependent way acting at local spinal circuits while rTMS probably promoted changes in the presynaptic inhibition of spinal motoneurons. In addition, the association of two neuromodulatory techniques induced long-lasting changes.

## Introduction

Repetitive transcranial magnetic stimulation (rTMS) and trans-spinal direct current stimulation (tsDCS) are safe and effective non-invasive tools involving the application of magnetic and electric fields to induce changes in the spinal cord and cortical excitability [1, 2].

In humans, rTMS post-stimulation effects are widespread to cortical and subcortical areas [3] and depends on frequency, site of stimulation and coil position [4]. Usually, high frequency rTMS (≥5Hz) increase corticospinal excitability while low frequency rTMS (<1Hz) has the opposite effect [1]. At spinal cord level, rTMS seems to induce changes in presynaptic inhibition in Ia afferents terminals; this way, few studies using high-frequency rTMS over the motor cortex showed a decrease of the Hoffmann reflex [5, 6] while low-frequency rTMS increased it [7].

tsDCS is another non-invasive tool capable of modulating central nervous system excitability through delivering a direct current (DC) over the spinal cord [2, 8]. Some preliminary studies suggest that anodal tsDCS depresses ascending spinal pathways conduction [8, 9] and induces a long-lasting decrease in post-activation depression of soleus H-reflex [10]. In animals, mice model, cathodal tsDCS increase corticospinal output through changes in neurotransmitters release at segmental level [11] and by the modulation of the corticospinal pathways conduction [12, 13].

As well as non-invasive stimulation tools, physical exercise modulates the cortical and spinal cord excitability [14]. The mechanisms related to the cortical modulation by physical exercise results from the decrease of intra-cortical inhibition and the increase of intracortical facilitation networks on primary motor cortex [15]. Alternatively, physical exercise acts at the spinal level inducing changes in the synaptic efficacy between Ia afferents and motoneuron connections [16]. In either site, cortex or spinal cord, the effects of physical exercises depend on intensity, duration and complexity of training [15–20].

Due to its ability to induce changes in the excitability of neural circuits and to promote neuronal plasticity, rTMS and tsDCS have been used in isolation as non-pharmacological method to treat drug addiction [21, 22], to reduce spinal cord reflexes [5, 6, 23, 24] and to maximize motor recovery in patients with neurological disorders [1, 25–28]. However, recent studies have pointed to the benefits of the combination of different non-invasive stimulation techniques as an adjunct intervention to augment the response of the motor system to the behavioral training [29, 30]. This concept is supported by the “metaplasticity” theory in which a previous neuronal activity modulates the capacity for subsequent plastic change through the induction of long-term potentiation (LTP) and depression (LTD) mechanisms [31].

Therefore, the induction of LTP or LTD in the central nervous system can vary as a function of the integrated postsynaptic activity. This way, the association of two neuromodulatory techniques could reinforce the effects of the first intervention or activate neuroprotective mechanisms to stabilize synaptic weights in neuronal networks in order to maintain synaptic plasticity (29). This latter includes the reversion of the “facilitatory preconditioning” as well as “inhibitory preconditioning” [32]. For this reason, to understand how the non-invasive neuromodulatory tools act when combined with behavioral approaches in healthy models is critical in providing basis for future therapeutic application in patients with neurological disorders.

The purpose of the present study is to clarify the effects of the association of two different non-invasive tools with the treadmill exercise on the spinal cord and motor cortex excitability in a triple blind, randomized and sham-controlled crossover study with healthy subjects.

## Materials and methods

A triple blind, randomized, sham-controlled study was performed in the Applied Neuroscience Laboratory of Physical Therapy department at Universidade Federal de Pernambuco. The study’s protocol was approved on June 02, 2016 by the local Ethics Committee conforms to the standards of the Declaration of Helsinki and all participants gave their written informed consent before being included in the study. The study protocol was registered in ClinicalTrials.gov (name: NIS_CNSexcitability, number: NCT02659826) and in Protocols.io (protocol DOI: dx.doi.org/10.17504/protocols.io.nkidcue). There was a delay in registering the trial because we were genuinely unaware of the importance of registration preceding the enrolment of participants. The authors confirm that all ongoing and related trials for this intervention are registered. All volunteers included into the study were recruited between June and December 2016.

### Participants

Twelve healthy volunteers (6 males; 24.75 ± 2.77 years) were recruited at Recife, Pernambuco, Brazil. As Inclusion criteria, we considered (i) age ranging between 18 to 40 years old; (ii) healthy volunteers (self-report); (iii) right handed, confirmed by Edinburgh Handedness Inventory [33] and (iv) sedentary or irregularly active according to brief version of International Physical Activity Questionnaire—IPAQ [34]. Only women controlling menstrual cycle through contraceptive medicine were included [35]. In contrast, exclusion criteria included: (i) metallic implant in the skull, face, spine or pacemaker; (ii) pregnancy; (iii) history of epileptic seizure; (iv) history of neurological, serious systemic or psychiatric disorders; (v) current use of anti-depressive or other medication that could change the cortical excitability.

The sample size was defined using the statistics calculator from the Massachusetts General Hospital Biostatistics Center (http://hedwig.mgh.harvard.edu/sample_size/js/js_crossover_quant.html). The minimum detectable difference (MDD = 1.96* Standard error*√2) and the standard deviation of the mean difference were calculated from previous studies, that assessed the Hoffman reflex amplitude after and before tsDCS or rTMS session in healthy subjects [2, 10]. We found a value of 765.92 for the minimum detectable difference. A statistical power of 80% and a two-sided significance level of 5% were assumed for all analysis.

### Experimental procedure

A counterbalanced and randomized session sequence was generated by computer (www.randomization.com). Each volunteer underwent six experimental sessions consisting of a baseline assessment of cortical and spinal excitability followed by a randomly assigned non-invasive stimulation (NIS) session, tsDCS (anodal, cathodal or sham) or TMS (20 Hz, 1Hz or Sham), tested in a double-blind condition. For this reason each volunteer was followed for six weeks. To guarantee the blinded condition, stimulator programming and electrode positioning were made by a different physician not involved in enrollment, cortical and spinal cord excitability assessment nor statistical analysis [24]. The allocation process was conducted by an external researcher and keeped in sealed envelopes. All assessors, volunteers, and statistician were blinded regarding the kind of non-invasive stimulation (triple-blind study).

All individuals were oriented to maintain the same feed and sleeping habits during the study. All intervetions were performed at the same shift of the day. In addition, feeding habits, motivation, amout of sleep, sleep quality and fatigue level were evaluated before the start of each session to guarantee the same basal condition. After the NIS session all subjects underwent to 20 minutes of treadmill training with moderate intensity following immediate (T0), 30 minutes (T30) and 60 min (T60) after stimulation re-assessment. At least an one week washout between sessions was adopted to avoid any influence by previous stimulations.

#### Trans-spinal direct current stimulation (tsDCS)

A direct current was delivered by an eletric stimulator (Neuroconn®/Germany) connected to a pair of electrodes (7 cm x 5 cm) of saline-soaked synthetic sponge placed between eleventh and twelveth thoracic vertebras (active electrode) and on right shoulder (reference electrode). The tsDCS polarity refered to the electrode placed over the spinal cord. The electrodes on the right shoulder and the spine were attached all at once and activated only according to the sequence indicated by the randomization procedure. Direct current was delivered for 1200s, fade in and fade off 10s with an intensity of 2.5 mA and a current density of 0.071 mA/cm^2^. Sham stimulation followed the same montage of anodal stimulation but after 30 seconds, the stimulator was turned off. The sham montage provided the same initial sensation of active stimulation but did not induce neurophysiological changes [36]. After tsDCS all subjects were interviewed about possible adverse effects [37].

#### Repetitive transcranial magnetic stimulation (rTMS)

rTMS was delivered by eight coil attached to a magnetic stimulator (Rapid Magstin®). All volunteers were seated in a comfortable chair with headrest and armrests. Before stimulation, a rest motor threshold (RMT) of the first dorsal interosseous (FID) muscle was determined on the left motor cortex. The RMT was defined as the lowest single-pulse TMS intensity required to produce a motor evoked potential amplitude larger than 50mV (confirmed by surface electromyography) [38]. For RMT assessment we used the *Motor Threshold Assessment Tool*—MTAT 2.0 (http://www.clinicalresearcher.org/software.htm). For high frequency repetitive stimulation, a total of 1800 pulses were delivered by two trains with two seconds of duration at a frequency of 20Hz (40 pulses/train) and 28 seconds of interval. For 1Hz rTMS, a total of 1500 pulses were delivered. For both active rTMS conditions, a repetitive stimulation was applied over the FID hotspot with an intensity of 90% of MT. For sham rTMS, a coil disconnected from the stimulator unit was held over the scalp while a second coil, connected with the stimulator, was positioned behind the patient’s head, without touching the scalp. Thus, no current was induced in the brain, but the subjects were exposed to acoustic stimulation from the second coil [38].

#### Treadmill exercise protocol

A treadmill walk (Gait training- Biodex) with moderate intensity (64% to 76% of maximum heart rate) and 20 minutes duration was performed after each NIS session. The maximum heart rate (HR_max_) was calculated using the following formula: HR_max_ = 220 –Age [39]. In order to guarantee similar exercise intensity for all volunteers, the subject perceived exertion and heart rate were assessed by Borg scale and monitored by cardiac polar (Polar RS800, Polar Electro- Kempele, Finland) every three minutes. Moreover, treadmill inclination and speed were controlled to avoid fatigue or light training.

#### Hoffmann reflex and homosynaptic depression

All subjects were positioned comfortably in a quiet room on lying position. The lower limbs were placed with knee flexed 30° and ankle at 90°. The Hoffmann reflex (Hr) was elicited and recorded by eletrical stimulator (Neuromep-8, Russia) and electric stimuli were delivered to the tibial nerve at popliteal fossa [9]. Surface electromyography was recorded by self-adhesive Ag–AgCl electrodes (1.0 cm diameter) with 2 cm interelectrode distance. Two electrodes were positioned on the medial portion of the right soleus, 5 cm and 7 cm distally to the medial head from gastrocnemius muscle [10]. A ground electrode was attached over gastrocnemius midline 10 cm distally to recorded electrodes.

To obtain the maximal Hr (Hrmax) and maximal M-wave (Mmax), rectangular pulses with 1.0 ms were delivery every 12 seconds and the current intensity increased in steps of 1 mA. Recordings were collected at a bandwidth of 5–10000 Hz and a sample rate of 20000 Hz and care was taken to keep impedance below 3 kΩ. The Hr_max_/M_max_ ration was considered during statistical analysis to minimize physiological variations.

Homosynaptic depression was assessed by H-reflex recovery curve [40–42]. We plotted the Hr recovery curve by delivering pairs of stimuli following the same setting described above with equal Hr_(max)_ intensity and duration. Stimuli were delivered at interstimulus interval (ISI) of 40, 50, 70, 75, 100, 150, 200, 250, 300, 350, 400, 450, 500, 600, 700, 800 and 900 ms, and 1, 2, 3, 4 and 5 seconds at frequency no faster than 12 seconds. The mean peak-to-peak amplitude of the unconditioned and conditioned H-reflex was calculated. Conditioned mean was expressed as a percentage of the unconditioned mean for every delay (Hr2/Hr1 x 100) [41].

#### Nociceptive flexion reflex (NFR)

The painful component of NFR was assessed in a quiet room (temperature 23 ± 2°C) with volunteers positioned on the left side to ensure complete muscle relaxation and the right leg rest adjusted to maintain knee flexion at 60° degrees from horizontal.

To elicit NFR, five consecutive electrocutaneous stimuli were delivered over the sural nerve through Neuro-Mep bar electrodes (a bipolar stimulation electrode with cathode placed on superior extremity) applied behind the right lateral malleolus. Each stimulation trial consisted of five rectangular pulses with 1ms duration and intensity equivalent to 120% of NFR threshold. To avoid the stimulus predictability the stimulation was delivered randomly (interval range 8 to 12 s) without previous warning [43]. To record NFR activity, a differential electromyographic pair of surface electrodes (Ag/AgCl, inter-electrode distance of 2 cm) was positioned on the belly of ipsilateral brevis head of the biceps femoris muscle of the right leg and a reference (common ground electrode) on **l**ateral epicondyle of femur [44]. The analysis time considered the post-stimulation interval of 90 ms to 150 ms. The EMG signals were amplified (up to 20 000 times) and the filtered, bandpass 5 Hz to 2 KHz (Neuro-MEP, Neurosoft®, Russia).

For the NFR threshold assessment, the current intensity was increased from 6 mA in steps of 1 mA until achieving a reflex with an amplitude exceeding 20 μV in the 90 to 150 ms after stimulation interval [43]. A perceived report of pain after each stimulation was recorded using Visual Analog Scale (VAS) that scores 0 (no pain) to 10 (maximum tolerable). In the event that a participant provided a rating of 10, the threshold assessment was discontinued [44].

#### Motor-evoked potential (MEP)

The corticospinal excitability was assessed by MEP elicited through transcranial magnetic stimulation. For this, the subjects were seated in a comfortable chair with head and arms resting. A figure-of-eight coil (7 cm diameter) connected to a magnetic stimulator (Neurosoft Ltd., Russia, peak magnetic field = 2.2 tesla) delivered the TMS single pulses. The coil was kept in a constant position centered over the motor cortex on left hemisphere at an angle of 45° from the midline. The optimal position (hotspot) was defined as the site where stimulation resulted the largest evoked potentials. MEP were recorded at rest by two non-polarizable surface electrodes (AgCl), one placed over the belly of the first dorsal interosseous (FDI) muscle, and the other on the skin overlying of the first metacarpophalangeal joint of the first finger of the right hand.

Stimulation was set as 130% of resting motor threshold (as previously described). To compare MEP modifications across the time we fixed the same stimulation intensity (baseline). Twenty MEPs were collected for each time point and all outputs were amplified and filtered (bandwidth 5Hz to 20 KHz, Neuro-MEP-Micro, Neurosoft Company, Russia).

### Statistical analysis

A descriptive analysis was performed to present clinical characteristics and one-way ANOVA (with post hoc LSD) was employed in order to verify any difference between conditions before each session.

For the analysis of spinal and cortical excitability measurements, the values obtained after stimulation were rescaling intra-individually, i.e post-baseline measurements were divided by the baseline measurement. For homosynaptic depression, we selected the follow interstimulus intervals (150, 200, 250 and 300 ms) as a potentiation peak of recovery from the H-reflex recovery curve [40]. All data met criteria of normality (Shapiro-Wilk test) and the sphericity was checked by Mauchly’s test and corrected by Greenhouse-Geisser test, if necessary.

For Hr max/ M max amplitude, NFR area, MEP amplitude and HD a repeated measure ANOVA (6 x 4) was calculated using within subject factors: time course (baseline, immediately, 30 minutes and 60 minutes after stimulation) and stimulation (anodal tsDCS, cathodal tsDCS, sham tsDCS, 20 Hz rTMS, 1 Hz and sham rTMS). When appropriate, post hoc analyses were performed with paired Student t-test. There is no correction for multiple comparisons.

The magnitude of effect was given by Cohen’s d effect-size test (comparison with sham stimulation). We considered as a small effect-size when d is equal or less than 0.5, a moderate effect-size when d is between 0.5 to 0.7 and a large effect-size when d is greater than 0.8. All data was analyzed using the Statistical Package for Social Sciences (version 20.0, SPSS Inc, Chicago IL, USA). A P value of < 0.05 was considered significant for all statistical analysis.

## Results

**Fig 1:**
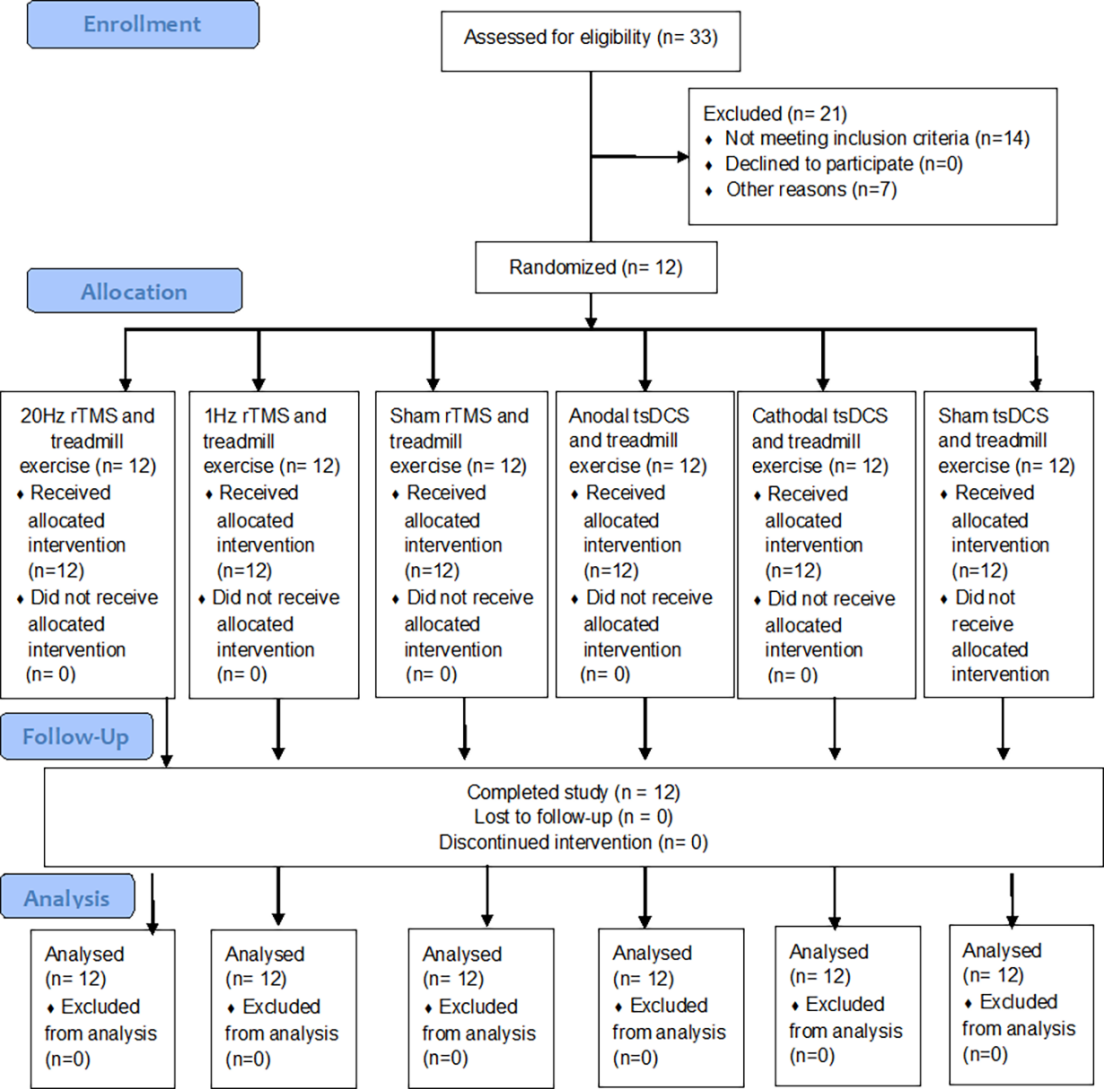
CONSORT flow diagram. TMS: Transcranial Magnetic Stimulation. tsDCS: trans-spinal Direct Durrent Stimulation.

Twelve healthy subjects (6 male, mean age 27.75 ± 2.77 years) completed the study. Three subjects were considered “sedentary” according to IPAQ (25%) and nine as “irregular active” (75%). In general, the experimental procedures were well tolerated by all subjects and only a few subjects reported an occasionally slight tingling or itching sensation beneath the electrodes. No difference was distinguishable between the “real” or “sham” stimulation nor between polarities in relation to sensations caused by stimulation (e.g., itching, tingling, or auditory perception). All participants underwent all stimulations and no subject dropped out of the study. The flow diagram of screening and selection process of individuals enrolled in the study are shown in Fig 1.

At baseline, one-way ANOVA revealed no difference between all six sessions for H_max_ / M_max_ amplitude (F = 0.33, p = 0.89), NFR area (F = 0.35, p = 0.88) and MEP amplitude (F = 0.21, p = 0.88), see Table 1. No difference was detected between the sessions in relation to feeding habits, motivation, amount of sleep, sleep quality and fatigue level at baseline assessment (S1 Table).

**Table 1:** Mean and standard deviation of the neurophysiological parameters at baseline (raw values).

Session	Hr max/M max amplitude (mV)	One-way ANOVA	MEP amplitude (mV)	One-way ANOVA	NFR area (nV*s)	One-way ANOVA
Sham tsDCS	50.62 ± 18.43	F = 0.33	0.99 ± 0.51	F = 0.21	498.92 ± 200.50	F = 0.35
Cathodal tsDCS	55.87 ± 16.60	p = 0.89	1.03 ± 0.67	p = 0.96	451.22 ± 302.24	p = 0.88
Anodal tsDCS	55.50 ± 17.75		1.04 ± 0.51		515.38 ± 160.89	
Sham rTMS	48.80 ± 15.95		1.15 ± 0.58		548.25 ± 285.13	
1Hz rTMS	50.88 ± 17.83		0.95 ± 0.44		480.19 ± 93.97	
20 Hz rTMS	53.02 ± 16.07		0.99 ± 0.39		447.78 ± 157.95	

Hr: Hoffmann Reflex. MEP: Motor evoked Potential. NFR: Nociceptive Flexion Reflex.

### H max / M max

**Fig 2:**
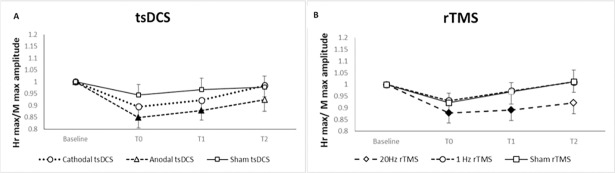
Induced effects of non-invasive stimulation combined with treadmill exercise in the monosynaptic reflex activity. A: Hr max/ Mmax amplitude ratio after trans-spinal direct current stimulation associated with treadmill. B: Hr max/ Mmax amplitude ratio after repetitive transcranial magnetic stimulation associated with treadmill. Shown are the mean ± SE of baseline-standardized Hr max /M max amplitude ratio before (baseline), immediately after (T0), 30 minutes (T30) and 60 minutes after experimental conditions (T60). Filled symbols indicate significant (p< 0.05) changes compared to baseline.

ANOVA for repeated measures disclosed a significant effect for factor TIME (F = 4.81, p = 0.007) for H max / M max amplitude. The post-hoc analysis performed with paired t-test revealed a significant decrease in Hr max / M max amplitude immediately (mean difference = 0.152; 95% CI = 0.06 to 0.25, p = 0.005) and 30 minutes (mean difference = 0.12; 95% CI = 0.04 to 0.21; p = 0.013) after anodal tsDCS combined with treadmill exercise (Fig 2A) and immediately (mean difference = 0.12; 95% CI = 0.04 to 0.26; p = 0.017) and 30 minutes (mean difference = 0.11; 95% CI = 0.10 to 0.21; p = 0.034) after 20Hz rTMS plus treadmill exercise compared to baseline values (Fig 2B).

### Nociceptive flexion reflex

The ANOVA for repeated measures showed a significant effect for interaction STIMULATION x TIME (F = 2.744, p = 0.009) for NFR area. The post-hoc analysis performed with paired t-test revealed a significant decrease in NFR area max immediately after anodal tsDCS/treadmill (mean difference = - 0.38; 95% CI -0.62 to—0.14; p = 0.004, d = 0.97) compared to sham tsDCS/treadmill.

Paired t-test also disclosed a significant increase of NFR area immediately (mean difference = - 0.90; 95% CI -1.27 to—0.53; p < 0.001), 30 minutes (mean difference = - 0.76; 95% CI -1.09 to—0.42; p = 0.001) and 60 minutes (mean difference = - 0.57; 95% CI -0.99 to—0.15; p = 0.014) after 20Hz rTMS/treadmill compared to baseline values and in comparison to sham rTMS/treadmill at T0 (mean difference = 0.88; 95% CI 0.45 to 1.31; p = 0.014, p = 0.001, d = 2.67), T30 (mean difference = 0.82; 95% CI 0.29 to 1.35; p = 0.007, d = 2.07) and T60 (mean difference = 0.66; 95% CI 0.17 to 1.16; p = 0.014, d = 1.46), see Table 2.

**Table 2:** Baseline-standardized values for motor evoked potential, maximal Hoffmann reflex and M-wave ration, nociceptive flexor reflex and homosynaptic depression after repetitive transcranial magnetic stimulation and trans-spinal direct current stimulation associated with physical exercise sessions.

	tsDCS			rTMS			Repeated measures ANOVA
	Sham	Anodal	Cathodal	Sham	20 Hz	1Hz	
**MEP (mV)**							
T0	1.16 ± 0.29	0.86 ± 0.27*	1.03 ± 0.29	1.03 ± 0.34	1.47 ± 0.29*	1.10 ± 0.39	Stimulation: F = 4.82; p = 0.01; power = 0.96
T30	1.14 ± 0.34	1.02 ± 0.37	1.15 ± 0.29	1.14 ± 0.39	1.59 ± 0.52*	1.12 ± 0.39	Time: F = 7.18; p = 0.01; power = 0.96
T60	1.18 ± 0.45	1.08 ± 0.37	1.15 ± 0.40	1.18 ± 0.40	1.54 ± 0.43*	1.15 ± 0.28	Stimulation vs Time: F = 1.72; p = 0.05, power = 0.90
**H max/ M max (mV)**							
T0	0.94 ± 0.16	**0.85 ± 0.15**	0.89 ± 0.18	0.92 ± 0.14	**0.88 ± 0.15**	0.93 ± 0.17	Stimulation: F = 1.22; p = 0.31; power = 0.40
T30	0.97 ± 0.17	**0.88 ± 0.14**	0.92 ± 0.16	0.97 ± 0.14	**0.89 ± 0.16**	0.97 ± 0.19	Time: F = 4.81; p = 0.007; power = 0.86
T60	0.98 ± 0.16	0.92 ± 0.17	0.98 ± 0.17	1.01 ± 0.17	0.92 ± 0.16	1.01 ± 0.15	Stimulation vs Time: F = 0.64; p = 0.83, power = 0.41
NFR (nV*s)							
T0	1.19 ± 0.35	0.89 ± 0.26 *	1.36 ± 0.35	1.03 ± 0.30	1.90 ± 0.51*	0.88 ± 0.39	Stimulation: F = 2.12; p = 0.15; power = 0.46
T30	1.01 ± 0.27	0.85 ± 0.23	1.24 ± 0.39	0.91 ± 0.34	1.76 ± 0.47*	0.94 ± 0.42	Time: F = 1.31; p = 0.35; power = 0.21
T60	0.96 ± 0.18	0.94 ± 0.35	1.17 ± 0.36 *	0.88 ± 0.31	1.57 ± 0.59*	0.93 ± 0.28	Stimulation vs Time: F = 2.74; p = 0.009, power = 0.95
**HD (mV)**							
**ISI 150 ms**							
T0	0.96 ± 0.27	1.03 ± 0.29	0.91 ± 0.24	0.94 ± 0.25	0.95 ± 0.27	0.83 ± 0.21	Stimulation: F = 0.48; p = 0.80; power = 0.17
T30	1.03 ± 0.26	1.11 ± 0.39	1.09 ± 0.37	0.97 ± 0.11	0.94 ± 0.24	0.98 ± 0.20	Time: F = 2.40; p = 0.12; power = 0.42
T60	0.91 ± 0.40	1.01 ± 0.32	1.05 ± 0.23	0.91 ± 0.28	0.91 ± 0.21	0.99 ± 0.16	Stimulation vs Time: F = 0.78; p = 0.07, power = 0.49
**ISI 200 ms**							
T0	0.84 ± 0.25	0.98 ± 0.20	1.00 ± 0.21	1.14 ± 0.37	1.01 ± 0.37	1.00 ± 0.20	Stimulation: F = 0.56; p = 0.73; power = 0.18
T30	0.93 ± 0.10	0.98 ± 0.26	0.95 ± 0.23	1.07 ± 0.23	0.99 ± 0.26	1.14 ± 0.32	Time: F = 0.91; p = 0.45; power = 0.26
T60	0.85 ± 0.26	0.94 ± 0.16	0.97 ± 0.21	0.90 ± 0.32	0.95 ± 0.21	1.02 ± 0.17	Stimulation vs Time: F = 0.94; p = 0.53, power = 0.58
**ISI 250 ms**							
T0	0.96 ± 0.26	1.01 ± 0.13	1.04 ± 0.22	0.97 ± 0.16	0.93 ± 0.20	1.05 ± 0.23	Stimulation: F = 1.10; p = 0.37; power = 0.22
T30	0.99 ± 0.20	0.84 ± 0.18	0.95 ± 0.18	1.03 ± 0.25	0.90 ± 0.26	1.06 ± 0.20	Time: F = 0.99; p = 0.42; power = 0.27
T60	1.02 ± 0.20	0.89 ± 0.23	0.94 ± 0.14	0.85 ± 0.32	1.01 ± 0.26	1.10 ± 0.19	Stimulation vs Time: F = 1.11; p = 0.36, power = 0.66
**ISI 300 ms**							
T0	0.89 ± 0.17	0.96 ± 0.19	0.83 ± 0.31	0.94 ± 0.26	0.87 ± 0.21	0.92 ± 0.26	Stimulation: F = 0.54; p = 0.74; power = 0.18
T30	1.00 ± 0.12	0.93 ± 0.20	0.99 ± 0.32	1.02 ± 0.21	1.00 ± 0.25	1.03 ± 0.22	Time: F = 2.48; p = 0.16; power = 0.30
T60	0.95 ± 0,07	0.97 ± 0.23	0.98 ± 0.18	0.92 ± 0.22	1.00 ± 0.29	1.04 ± 0.24	Stimulation vs Time: F = 0.79; p = 0.70, power = 0.48

Reported values for mean ± SD of baseline-standardized for motor evoked potential (MEP), maximal Hoffmann reflex and M-wave ration (H max/ M max) amplitude, nociceptive flexion reflex (NFR) area and homosynaptic depression (HD) amplitude, immediately after (T0), 30 minutes (T30) and 60 minutes (T60) after non-invasive stimulation associated with physical exercise. Bold numbers indicate significant (p < 0.05) changes compared to baseline. ISI: Interstimulus interval.

*p < 0.05 vs. sham condition.

### Homosynaptic depression

No statistical difference was found between mean values in any condition for the analysis over the time and in comparison to sham stimulation for interstimulus interval of 150 ms, 200 ms, 250 ms, and 300 ms (Table 2).

### Motor evoked potential

**Fig 3:**
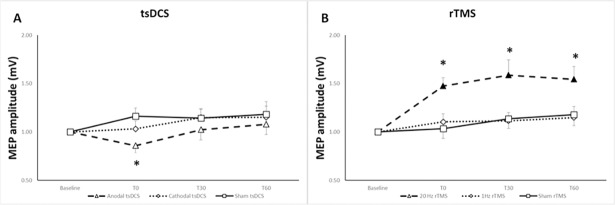
Induced effects of non-invasive stimulation combined with treadmill in the corticospinal excitability. **A:** MEP amplitude after trans-spinal direct current stimulation associated with treadmill. **B:** MEP amplitude after repetitive transcranial magnetic stimulation associated with treadmill. Shown are the mean ± SE of baseline-standardized MEP amplitude before (baseline), immediately after (T0), 30 minutes (T30) and 60 minutes after experimental conditions (T60). Filled symbols indicate significant (p< 0.05) changes compared to baseline. *p < 0.05 compared to sham condition.

The ANOVA for repeated measures disclosed a significant effect for factor STIMULATION (F = 4.82, p = 0.01) and TIME (F = 7.18, p = 0.01) for MEP amplitude. The post-hoc analysis performed with paired t-test revealed a significant decrease in MEP amplitude immediately after anodal tsDCS/treadmill (mean difference = - 0.30; 95%CI -0.57 to—0.04; p = 0.028, d = 1.07) compared to sham tsDCS/treadmill (Fig 3A).

Additionally, a significant increase of MEP amplitude was seen immediately (mean difference = - 0.32; 95% CI -0.55 to 0.08; p = 0.014), 30 minutes (mean difference = - 0.54; 95% CI -0.81 to—0.27; p = 0.001) and 60 minutes (mean difference = - 0.45; 95% CI = -0.73 to—0.17; p = 0.005) after 20Hz rTMS/treadmill compared to baseline values and in comparison to sham rTMS/treadmill at T0 (mean difference = 0.39; 95% CI 0.17 to 0.76; p = 0.042, d = 1.39), T30 (mean difference = 0.48; 95% CI 0.08 to 0.87; p = 0.024, d = 0.98) and T60 (mean difference = 0.42; 95% CI 0.02 to 0.82; p = 0.044, d = 0.87), see Fig 3B.

## Discussion

As far as we know, this is the first triple blind study that investigated the effects of different parameters of rTMS and tsDCS in combination with physical exercise on the spinal cord and corticospinal excitability. The main result of this study is that anodal tsDCS plus treadmill induced a reduction of MEP’s amplitude and NFR area compared to sham stimulation/treadmill and reduced Hr max/ M max ratio across the time up to 30 after stimulation offset. Conversely, cathodal tsDCS plus treadmill increased NFR area compared to sham group. In addition, 20 Hz rTMS plus treadmill strongly increased MEP’s amplitude and NFR area at least 60’ offset stimulation in comparison to sham stimulation/treadmill and decreased Hr max/ M max ratio across the time up to 30 after stimulation offset. No changes were found for homosynaptic depression values after any sessions for all interstimulus intervals tested.

Our results demonstrate that tsDCS and rTMS modulate monosynaptic and polysynaptic spinal reflexes through a different mechanism. tsDCS seems to concentrate its effects at a segmental level in dependence on polarity. On the other hand, the modulation of spinal reflexes by rTMS appears to be dependent of pre-synaptic inhibition/ facilitation of spinal motoneurons by descending outputs. The following topics discuss some possible mechanisms involved in the modulation induced by the combination of noninvasive stimulation techniques and physical exercise in spinal cord reflexes behavior and corticospinal excitability.

### Reduction of Hr max/M max after anodal tsDCS and rTMS combined with treadmill

Few previous studies using direct current stimulation in isolation over spinal cord reported no changes on Hoffmann reflex parameters after anodal tsDCS [9, 10, 45]. Even if the complete understanding about how tsDCS influences the spinal cord circuits remains open to question, some mechanisms have been suggested. The more widely accepted concept is that tsDCS does not have a significant influence on the excitability of the alpha-motoneuron but on the efficacy of the Ia fibre-motoneurone synapse [10].

Our study, unlike the above mentioned, observed a significant decrease across the time in H max/M max amplitude ration after anodal tsDCS and 20Hz rTMS followed by 20 minutes of moderate physical exercise session. The combination of these two neuromoduladory techniques forms the core aspect that differentiates our results from those that applied tsDCS or rTMS isolated.

Previous studies with moderate intensity exercises found a reduction of Hoffmann reflex up to 30 minutes after training [46, 47]. This change coming from inhibitory mechanisms within the central nervous system and from the activation of the afferent mechanoreceptors of the exercising leg [47]. Peripheral influences promoted by physical exercise include the effect of type III e IV afferent mechanoreceptors on pre-synaptic inhibition of the Ia afferent terminals, alpha motoneurons inhibition through Ib inhibitory neurons and reciprocal inhibition caused by activation of Ia afferent fiber from antagonist muscles like anterior tibialis [47, 48]. Therefore, the addition of anodal tsDCS and 20 Hz rTMS increased the effects of exercise and extended it up to 30 minutes after intervention. This finding could be result of induced plastic changes at the spinal level.

The mechanisms underlying plastic changes on spinal circuits induced by tsDCS are not fully understood, but some inferences based on transcranial DC stimulation suggest that changes on resting membrane potential acts similarly to long-term potentiation (LTP) and long-term depression (LTD) mechanisms [10, 36]. Spinal DC stimulation also mediates changes on the glutamatergic neurotransmission at spinal level and influences motor cortical outputs in a polarity-dependent fashion [49].

Regarding the effects of the association of 20 Hz rTMS and treadmill exercise, our results corroborate with previous studies that assessed the spinal cord excitability after high frequency rTMS in isolation [5, 6]. Perez and colleagues (2005) demonstrated an increase of cortical motor evoked potential and a depression of soleus H-reflex after a 5 Hz rTMS single session. The authors suggested that the inhibition of H-reflex could be explained by an increase of presynaptic inhibition of soleus Ia afferent without changes on disynaptic reciprocal inhibition from antagonist muscles [6]. Therefore, high-frequency rTMS modulates specific spinal circuits’ transmission through changes on cortical drive even when the intensity of stimulation is below the threshold for elicitation of MEP [6].

### No changes in homosynaptic depression

In our study, no changes on homosynaptic depression were demonstrated after rTMS or tsDCS sessions. This result could be explained by the low complexity of the treadmill walking. Changes on synaptic efficacy have only been reported in studies that investigated the effects of complex training over spinal excitability [16, 19].

Mazzocchio and colleagues (2006) verified that constant cycling speed despite changing pedal resistance was associated with a persistent downregulation of the soleus H reflex, which was absent after cycling at the same speed without change in pedal. Similar results demonstrated an increase of homosynaptic depression immediately and up to three days following skilled cycle training [16] or visuo-motor task training [19] while no changes on non-skilled training were reported. These studies indicates that only skilled training is able to increase a presynaptic inhibition of Ia aferentes through changes of the probability of transmitter release without modify somatosensory ascending pathways [16, 19]. Thus, the spinal cord is able to express use-dependent plasticity at segmental level according to motor task complexity. As previously stated, treadmill walking is considered a non-skilled training due a constant speed and the absence of challenge during intervention.

### Increase of NFR after 20Hz TMS and cathodal tsDCS and decrease after anodal tsDCS stimulation

A depression of the ascending nociceptive conduction in spinal pathways after anodal tsDCS has been previous reported [9, 24, 50]. In these studies, tsDCS was applied in isolation and its inhibitory effects on ascending transmission remained at least 30 minutes after stimulation offset. Our study presented similar results after the session with anodal tsDCS combined with treadmill exercise. We also found an increase of the NFR area after a session containing cathodal tsDCS following treadmill exercise.

Cogiamanian and colleagues (2011) verified a lasting after-effects induced by anodal tsDCS on the central nociceptive transmission without changes on mono-oligosynaptic segmental reflex pathways. The authors suggested that tsDCS reduced NFR area by acting at the spinal level through changes in a complex interneuronal network that includes multireceptive and wide-dynamic-range (WDR) neurons [9]. WDR integrate a descending motor drive and multisensorial feedback in order to project their outputs to spinal motoneurons [51]. Therefore, anodal tsDCS in isolation or combined with exercise may act by reducing the gain on spinal nociceptive transmission through changes in interneuron spinal networks while cathodal tsDCS exerts the opposite effect.

Other mechanisms has been related with NFR inhibition by tsDCS, such as changes on neurotransmitters release (especially GABA and glutamate) at spinal cord level [52] and the activation of supraspinal loops with brain stem and thalamocortical system that provides descending inhibition commands to the spinal motoneurons [53]. In addition, changes in NMDA (N-metil D-Aspartato) receptors efficiency at segmental and intersegmental level by tsDCS could block the “gain” of nociceptive transmission [24].

Regarding the effects of rTMS in the nociceptive flexion reflex, previous studies reported no changes in the NFR size and threshold after a single session of 10 Hz rTMS over the motor cortex [54, 55]. However, there is some evidence that sensory thresholds, like pain threshold for cold and heat sensation, can be modulated by rTMS [56, 57]. Therefore, the use of rTMS in isolation could induce changes in the ascending spinal conduction without an effective activation of the descending modulatory system [55, 57].

In our study, we demonstrated for the first time an increase of NFR area after the association between high-frequency rTMS and treadmill. The effect of moderate intensity exercise in the NFR has not been fully understood; however, Hosseinzadeh and colleagues (2013) described a significant acute decrease of NFR threshold after a bout of unaccustomed high-intensity exercise. The authors suggested that exercise induced a central sensitization due to the facilitation of the sensory component of the reflex arc after performing the exercise [58].

Even if our exercise protocol has some divergences in comparison to above-mentioned study, we also speculate that treadmill activated other afferents inputs in the reflex pathways as mechanoreceptors, joint, cutaneous nociceptive and stretch receptors afferents [58]. All these multisensorial inputs converge onto common interneurons in the spinal cord and, together with the enhancement of excitatory drive from the corticospinal tract, could induce an increment of the nociceptive reflex responses [53].

### Changes on MEP’s amplitude after session of 20Hz rTMS and anodal tsDCS combined with treadmill

Our results demonstrated a reduction of MEP’s amplitude immediately after experimental session containing anodal tsDCS/treadmill compared to sham tsDCS/treadmill session. Additionally, a session containing 20 Hz rTMS associated with treadmill induced a significant MEP increase compared to sham rTMS/treadmill session and to the baseline condition.

The mechanisms with regard to how tsDCS influences the corticospinal excitability remains hypothetical. However, some concepts from studies that assessed the cortical effects of tsDCS in isolation in animal and human might help to discuss our findings. Ahmed and Wierasko (2012) demonstrated that tsDCS modulates corticospinal output through changes in neurotransmitters release in the spinal cord of rats [11]. The same author verified that cathodal tsDCS amplifies segmental responses to cortical drive through the increasing of glutamate release and blocking of the GABA receptors at segmental level in mice [52]. Moreover, recent evidence indicates that tsDCS modulates the transmission in ascending spinal pathways and their cortical target in rats [12] and changes the conductive properties of corticospinal tract through decreasing the number of axons conducting the evoked potential [13].

Concerning the enhancement in MEP’s amplitude following high-frequency rTMS/treadmill, our study confirmed the long lasting effects induced by a stimulation with intensities below resting motor threshold. These findings corroborate with previous reports using high-frequency rTMS in isolation [59, 60]. We also expand previous knowledge about the rTMS’s ability to activate other regions of the primary motor cortex not directly related to stimulation once the increment of FDI motor evoked activity was seen after 20 Hz rTMS delivered over the cortical representation of lower limbs.

The enhancement of corticospinal excitability supports the notion that long period (> 900 stimuli) subthreshold high-frequency rTMS increases transmission in synaptic connections to pyramidal cells on motor cortex [61]. This alteration might occur due to the activation of later waves, I waves, and by the decrease of intracortical inhibition mechanisms in the primary motor cortex [62, 63]. Considering that high frequency rTMS can enhance practice-dependent plasticity [64], the association between 20 Hz rTMS and treadmill used in this study induced a lasting increase in MEP amplitude up to 60 minutes offset stimulation. The prolonged effects may be caused by the inducement of plastic changes in neuronal circuits that potentiated synaptic connections on pyramidal cells in M1 or between pyramidal cells and spinal motoneurons [61].

### General remarks

The strengths of this study include the following (a) methodological rigor during patient’s selections, allocation concealment, random sequence generation and blinding of participants; (b) the use of independent and masked assessors and statisticians during study’s conduction and data analysis; (c) the use of appropriate wash out interval between the sessions that avoided carry over effects; (d) a clear and transparent data report and (e) an unprecedented comparison between the effects of different montages of rTMS and tsDCS combined with treadmill in the spinal cord and corticospinal excitability.

The main limitation of this study relates to the lack of a session containing only tsDCS or rTMS. This absence hampers the comparison between our results with previous studies that applied tsDCS or rTMS in isolation. Moreover, an extra comparison would help to elucidate differences between the pure effects of the neuromodulatory techniques and potential changes due to metaplasticity mechanisms induced by the association with the treadmill. Another limitation was no correction for multiple comparisons has been performed, increasing the likelihood of type I errors.

In conclusion, our data provide evidence that a single session of tsDCS associated with treadmill modulates the spinal cord excitability in a polarity-dependent way. We showed that anodal tsDCS/treadmill decreases the spinal cord excitability (as the monosynaptic and polysynaptic reflexes) and motor evoked responses while cathodal tsDCS/treadmill increases the polysynaptic reflex. In addition, we demonstrated that 20 HZ rTMS/treadmill reduce monosynaptic reflexes through the increment of descending modulatory system activity but increase polysynaptic responses (nociceptive flexion reflex). In both conditions, the neuromodulatory effects remained up to 60 minutes after stimulation offset, these long-lasting effects could be related to synaptic plasticity induced by the combination of tsDCS and rTMS with the treadmill.

## Supporting information

S1 Table Description of feed habits, motivation, amount of sleep and fatigue level at baseline. (DOCX)

S1 Text CONSORT checklist. (DOC)

S2 Text Study protocol. (DOCX)

S3 Text Ethics committee approval. (PDF)
