# Supplementary material for: Effects of repetitive transcranial magnetic stimulation and trans-spinal direct current stimulation associated with treadmill exercise in spinal cord and cortical excitability of healthy subjects: A triple-blind, randomized and sham-controlled study
Source: PLoS One. 2018 Mar 29;13(3):e0195276. doi: 10.1371/journal.pone.0195276 (PMC5875883; doi:10.1371/journal.pone.0195276)
Supplement: S1 Table — (DOCX) [file pone.0195276.s001.docx]

|  | **Sham tsDCS** | **Anodal tsDCS** | **Cathodal tsDCS** | **Sham rTMS** | **20 Hz rTMS** | **1Hz rTMS** | **p-valor**  (One-way ANOVA) |
| --- | --- | --- | --- | --- | --- | --- | --- |
| **Feeding habits - n (%)** |  |  |  |  |  |  |  |
| Intake of stimulant foods and drinks (e.g. coffee. tea. energy drinks) | 4 (33.33) | 6 (50.00) | 4 (33.33) | 5 (41.66) | 2 (16.66) | 4 (33.33) | 0.66 |
| **Motivation - n (%)** |  |  |  |  |  |  |  |
| Session |  |  |  |  |  |  |  |
| Not motivated | - | 1 (8.33) | - | - | - | - |  |
| Poorly motivated | 1 (8.33) | 2 (16.67) | 2 (16.67) | - | 1 (8.33) | 1 (8.33) |  |
| Middling motivated | 7 (58.33) | 4 (33.33) | 4 (33.33) | 6 (50.00) | 5 (41.67) | 6 (50.00) | 0. 88 |
| Very motivated | 3 (25.00) | 4 (33.33) | 4 (33.33) | 5 (41.67) | 6 (50.00) | 3 (25.00) |  |
| Extremely motivated | 1 (8.33) | 1 (8.33) | 2 (16.67) | 1 (8.33) | - | 2 (16.67) |  |
| Week |  |  |  |  |  |  |  |
| Not motivated | - | - | - | - | - | - |  |
| Poorly motivated | 1 (8.33) | - | - | 1 (8.33) | 1 (8.33) | - |  |
| Middling motivated | 6 (50.00) | 8 (66.67) | 7 (58.3) | 4 (33.33) | 6 (50.00) | 3 (25.00) | 0.48 |
| Very motivated | 5 (41.67) | 4 (33.33) | 3 (25.00) | 5 (41.67) | 5 (41.67) | 8 (66.67) |  |
| Extremely motivated | - | - | 2 (16.67) | 2 (16.67) | - | 1 (8.33) |  |
| **Amount of sleep in minutes** (mean ± SD) | 397.51 ± 58.79 | 395.00 ± 58.39 | 437.52 ± 48.64 | 427.51 ± 62.83 | 437.51 ± 79.33 | 402.52 ± 68.24 | 0.19 |
| **Sleep quality** (median) | 7 | 8 | 7.5 | 8 | 8 | 7 | 0.35 |
| **Fatigue** **level** (median) | 5 | 5 | 4.5 | 3 | 4.5 | 5 | 0.13 |
