# Supplementary material for: Effects of repetitive transcranial magnetic stimulation and trans-spinal direct current stimulation associated with treadmill exercise in spinal cord and cortical excitability of healthy subjects: A triple-blind, randomized and sham-controlled study
Source: PLoS One. 2018 Mar 29;13(3):e0195276. doi: 10.1371/journal.pone.0195276 (PMC5875883; doi:10.1371/journal.pone.0195276)
Supplement: S2 Text — (DOCX) [file pone.0195276.s003.docx]

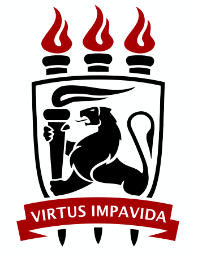
**UNIVERSIDADE FEDERAL DE PERNAMBUCO**

**CENTRO DE CIÊNCIAS DA SAÚDE**

**PROGRAMA DE PÓS-GRADUAÇÃO EM NEUROPSIQUIATRIA E CIÊNCIAS DO COPORTAMENTO**

**Projeto principal:** Estimulação não invasiva do sistema nervoso central associada ao treino locomotor com suporte de peso corporal na recuperação de pacientes lesados medulares.

**Subprojeto:** Efeito de diferentes tipos de estimulação não invasiva do sistema nervoso central sobre a excitabilidade cortical, medular e na percepção da dor de indivíduos saudáveis.

**Discente/Pesquisador:** Plínio Luna de Albuquerque

**Orientadora:** Profª. Dr^a^. Kátia Karina do Monte Silva

**CAAE:** 41231715.6.0000.5208

RECIFE-PE

Janeiro-2015

# Desenho do estudo

Trata-se de um estudo crossover, duplo-cego, sham-controlado, pseudorandomizado e contrabalanceado.

# Local do estudo

O estudo será realizado no Laboratório de Neurociência Aplicada (LANA) da Universidade Federal de Pernambuco (UFPE).

1. **Período do estudo**

A previsão de duração do estudo é de março 2015 a novembro de 2016.

1. **População do estudo**

A população do estudo será composta por voluntários saudáveis.

1. **Amostra**

**5.1 Amostragem**

Os voluntários serão recrutados por meio de anúncios em meios eletrônicos no

Campus da Universidade Federal de Pernambuco, selecionados e convidados a participar do estudo.

## 5.2 Tamanho amostral

Para o cálculo do tamanho amostral foi utilizado o software GPower, versão 3.1.3 para Windows (Franz Faul, Universität Kiel, Germany). O cálculo foi realizado de acordo com as médias e desvios padrões da amplitude do reflexo de retirada dos grupos do estudo de Cogiamanian et al. (2011). O nível de significância (α) adotado para o cálculo da amostra foi de 0,05 e o poder (β) adotado foi de 0,80.

## 5. 3 Critérios de Elegibilidade

A amostra será composta por indivíduos que atendam os seguintes critérios de

inclusão: (i) indivíduos que se auto relatem saudáveis; (ii) de ambos os sexos (iii) na faixa etária de 18-50 anos. Serão excluídos aqueles que possuam implante metálico ou qualquer outro dispositivo metálico no corpo; gravidez; histórico de crises convulsivas e epilepsia; presença de marcapasso e desordens neurológicas. Os voluntários que estejam fazendo uso de medicações ansiolíticas, antidepressivas ou qualquer outro medicamento que altere o nível de excitabilidade cortical são excluídos.

1. **Critérios para descontinuação do estudo**

Serão descontinuados do estudo os indivíduos que relatarem algum desconforto durante os procedimentos ou que não quiserem mais participar do estudo em qualquer momento da coleta de dados.

# Procedimentos experimentais

**7.1 Randomização e sigilo de alocação**

Para randomização, será utilizada uma tabela randômica gerada por programa de computador que determinará por sujeito a ordem das sessões com as técnicas de estimulação. Se necessário, parte da randomização poderá ser alterada (pseudo-randomizada), de modo a permitir que a ordem das sessões sejam contrabalanceada entre os voluntários.

A ordem das sessões de cada sujeito será codificado e a alocação será transferida para uma série de envelopes opacos numerados aos voluntários selecionados para o estudo. Os envelopes serão encaminhados ao pesquisador 2, responsável pela fase de aplicação da estimulação, ficando o pesquisador 1 cego quanto a técnica de estimulação que o voluntário será submetido.

- 1. **Avaliação inicial**

Inicialmente, os sujeitos elegíveis, após conceder a autorização para participação no estudo através do Termo de consentimento livre e esclarecido, serão convidados a responder um questionário semi-estruturado com dados epidemiológicos (idade, gênero, estado civil, grau de instrução, etc). Todos os voluntários também serão questionados sobre a ingestão de alimentos contendo cafeína (quantidade, frequência e tipo de alimentos) habitual durante as últimas 24 horas, medicação em uso e quantidade de horas de sono. Para as mulheres, a data da última menstruação será identificada.

**7.3 Estimulação não invasiva do sistema nervoso central**

Após a etapa da avaliação inicial, os indivíduos serão convidados a comparecer cinco vezes ao laboratório. Em cada visita, separada por no mínimo quarenta e oito horas, o voluntário será submetido a uma das seguintes técnicas de estimulação: (i) ETCCm catódica; (ii) ETCCm anódica; (iii) ETCCsham; (iv) EMTr-20Hz; EMTr 1Hz e (v) EMT-sham.

Para a ETCCm, uma corrente direta com intensidade de 2mA será aplicada durante 20 minutos através de um estimulador elétrico (modelo/marca: Neuroconn/Alemanha) conectado a dois eletrodos de dimensões 5 x 7 cm (35cm^2^) revestidos por uma esponja sintética e embebidos em solução salina. Na ETCCm anódica, o eletrodo ânodo será posicionado sobre o processo espinhoso da décima vértebra torácica (T10) e o eletrodo cátodo posicionado sobre o ombro direito. Na ETCCm catódica, o eletrodo cátodo será posicionado sobre T10 e o ânodo sobre o ombro direito. No grupo que receberá a estimulação sham os eletrodos serão posicionados nos mesmo locais da ETCCm ativa, no entanto, o estimulador será desligado após 30s de estimulação. Dessa forma, os pacientes experimentam as sensações iniciais no local estimulado, sem, no entanto, induzir efeito. O aparelho de eletroestimulação é desligado sem a percepção do paciente ([COGIAMANIAN et al., 2011](#_ENREF_2)).

Para a EMTr, bursts de 2s numa frequência de 20Hz (40 pulsos/trem) com um intervalo entre os trens de 28s, perfazendo um total de 1800 pulsos serão aplicado sobre M1 com uma intensidade de saída do estimulador equivalente a 90% do limiar motor de repouso (LMR) ([BENITO et al., 2012](#_ENREF_1)). A estimulação repetitivede baixa frequência (EMr1Hz) será compost por um total de 1500 pulsos entregues a uma frequência de 1Hz e uma intensidade equivalente a 90% do LMR. O LMR será considerado a menor saída do estimulador suficiente para obter um valor de potencial evocado motor (PEM) maior que 50μV de amplitude pico-a-pico em pelo menos 5 de 10 estimulações consecutivas. A estimulação magnética será aplicada através de uma bobina (70mm) conectada a um estimulador magnético (modelo/marca: Magstim Rapid/Reino Unido). Após a aplicação da estimulação, os voluntários serão submetidos a um treino de marcha na esteira.

Após cada sessão de ETMCC ou EMTr todos os indivíduos serão submetidos a 20 minutos de caminhada na esteira com intensidade moderada, seguido de reavaliações das variáveis neurofisiológicas no período imediatamente após (T0), 30 minutos (T30) e 60 minutos (T60) após a intervenção. Para evitar influência das estimulações prévias, será obedecido uma semana de intervalo entre as sessões.

# 7.4 Exercício na esteira

O treino de marcha será realizado sem suporte de peso em uma esteira (*Gait training- Biodex*) por 20 minutos com intensidade moderada (64% a 76% da frequência cardíaca máxima (FCmax) do indivíduo. A FCmax será definida através da fórmula: 220 – idade do voluntário. A intensidade dos exercícios foi estabelecida de acordo o guia para prescrição de exercício do *American College of Sports Medicine* ([GARBER *et al.*, 2011](#_ENREF_4)). Para garantir uma intensidade de exercício similar entre os indivíduos, todos os voluntários serão avaliados quanto a percepção subjetiva do esforço pela escala de Borg e monitorados por um polar cardíaco (Polar RS800, Polar Electro- Kempele, Finlândia). Adicionalmente, a inclinação e velocidade da esteira serão controlados para evitar fatiga ou esforço demasiadamente leve.

# 7.5 Avaliação da excitabilidade cortical

Para avaliação da excitabilidade corticoespinal será utilizado o Neuro-MS. A avaliação da atividade elétrica cerebral será realizada através da estimulação magnética transcraniana por pulso simples (EMT-p) e por pulso pareado (EMT-pp). Com a EMT pode-se realizar diferentes medidas para o estudo da excitabilidade cortical. A análise da excitabilidade do córtex motor será realizada através da: (i) determinação do limiar motor de repouso e (ii) amplitude do potencial evocado motor (PEM).

Os voluntários serão instruídos a sentar em uma cadeira e buscar uma posição confortável. Inicialmente estímulos simples de EMT serão administrados sobre o córtex motor para determinar a área de representação cortical do músculo primeiro interósseo dorsal (PID) - região cuja resposta de PEM se dá de forma mais intensa, observada através de eletromiógrafo. Os eletrodos, com diâmetro de 10 mm deverão ser colocados sobre o ventre do músculo alvo, e o eletrodo de referência será posicionado na articulação interfalangeana do polegar. Todos os cuidados para a aquisição do sinal eletromiográfico serão tomados de acordo com os critérios do Surface Electromyography for the Non-invasive Assessment of Muscles (SENIAM) ([HERMENS et al., 2000](#_ENREF_5)).

Para a localização da região cortical representativa do músculo PID, a bobina com uma angulação de 45 graus, será posicionada a uma distância de 20% do ponto Cz (segundo o sistema internacional de marcação 10-20), em direção ao trago contralateral ao músculo a ser avaliado ([PELLICCIARI ; BRIGNANI & MINIUSSI, 2013](#_ENREF_8); [LIU & AU-YEUNG, 2014](#_ENREF_6)). Durante a avaliação será utilizado uma bobina angulada em formato de oito, a fim de evitar viés de aferição. Serão realizadas as seguintes medidas:

1. **Determinação do limiar motor -** Para o limiar motor de repouso (LMR), será considerada a mínima intensidade da saída do estimulador necessária para emitir 5 pulsos de 10 com amplitudes acima de 50 microvolts. Para determinação do LMR será utilizado o software Motor Threshold Assessment Tool - MTAT 2.0 (http://www.clinicalresearcher.org/software.htm).
2. **Estudo do potencial evocado motor (PEM)-** Para determinação do PEM, a intensidade do estimulador magnético será ajustada para 130% do LMR, com o sujeito completamente relaxado, no qual 20 estímulos serão registrados. Serão observadas a média das amplitudes dos potenciais evocados para a determinação do PEM médio ([FILIPOVIC ; ROTHWELL & BHATIA, 2010](#_ENREF_3)).

# 7.6 Avaliação da excitabilidade medular

Para a avaliação da excitabilidade do segmento medular será utilizado o estimulador elétrico Neuromep-8, Rússia. As medidas referentes ao reflexo H (rH) e depressão homossináptica (DH) serão captadas por meio de eletrodos fixados no músculo sóleo. As medidas referentes ao reflexo de retirada da perna (RRP) serão captadas a partir da atividade serão obtidas a partir da captação da atividade muscular da cabeça curta do bíceps femoral do mesmo lado do estímulo.

Todos os pacientes serão orientados a se posicionar confortavelmente na posição deitada para com joelhos fletidos a 90º e tornozelos a 30º. Serão aderidos eletrodos de superfície (Ag-Cl), posicionados na porção medial do músculo sóleo a 5 cm da cabeça medial do músculo gastrocnêmio. O eletrodo terra será aderido no maléolo lateral. O eletrodo ânodo será posicionado na fosse poplítea e o cátodo próximo a patela ([WINKLER ; HERING & STRAUBE, 2010](#_ENREF_9)). Os sinais do reflexo-H serão obtidos por meio de um pulso simples retangular de 1ms sobre o nervo tibial disparado pelo eletrodo ânodo.

Para capitação do reflexo de retirada da perna (RRP), todos os voluntários receberão a mesma orientação da coleta do reflexo-H quanto ao posicionamento do corpo. O RRP será registrado a partir da liberação de um estímulo elétrico percutâneo no nervo sural por meio de um eletrodo de superfície posicionado atrás do maléolo lateral. Será registrada da atividade muscular do bíceps femoral da perna do mesmo lado. O estímulo para a deflagração do RRP consistira de um trem com 5 pulsos (duração de 1ms, frequência 200Hz) disparados aleatoriamente entre 5 - 20s ([COGIAMANIAN et al., 2011](#_ENREF_2)). Os registros serão sempre realizados no mesmo momento do dia para evitar influências do ritmo circadiano.

Os componentes relacionados às medidas do reflexo-H e reflexo de retirada da perna encontram-se descritos abaixo:

- Reflexo-H: O estímulo liberado será entregue por meio de uma corrente elétrica bipolar constante. Para obtenção da máxima amplitude do reflexo H e da onda M, serão entregues pulsos retangulares com 1ms de duração a cada 12 segundos. A corrente será aumentada a cada 1mA para obtenção do Limiar do reflexo-H (LrH), reflexo-H máximo, onda-M máxima ([COGIAMANIAN et al., 2011](#_ENREF_2)). A partir destes componentes serão analisados os valores referentes ao limiar (LrH) (mA), tempo de latência (ms) e amplitude (mV) do reflexo-H, além da razão Hmáximo/Mmáxima. Todos os registros serão coletados entre as bandas 5–10000 Hz e uma taxa de amostragem de 20000 Hz. Para minimizar artefatos fisiológicos, a impedância será mantida abaixo de 3 kΩ.
- Reflexo de retirada da perna (RRP): A intensidade de corrente necessária para evocar o estímulo será entregue a cada 2,5 mA até alcançar os limiares RIIr (componente inicial do reflexo, referente a sensibilidade tátil) e RIIIr (componente tardio referente a transmissão de estímulos dolorosos). O valor do limiar será considerado quando três registros consecutivos fornecerem o mesmo valor para o limiar. Para a captação da área total do RRP (mVms) e latência será aplicado um estímulo supralimiar (120% do limiar de RIIIr) e será considerada uma média de 5 registros consecutivos ([COGIAMANIAN et al., 2011](#_ENREF_2)). Os valores referentes ao limiar, latência e área serão analisados para o RRP total e para os componentes RIIr e RIIIr.
- Depressão homossináptica (DH): Para a determinação da DH, será realizado uma curva de recuperação. Para isto, pares de estímulos com a intensidade necessária para evocar um reflexo de Hofmman de máxima amplitude serão liberados nos seguintes intervalos interestímulos: 40, 50, 70, 75, 100, 150, 200, 250, 300, 350, 400, 450, 500, 600, 700, 800 e 900 ms, e 1, 2, 3, 4 e 5 segundos. Os pares de estímulos serão entregues com um intervalo de pelo menos 12 segundos entre eles. Para fins de análise será considerado a média da amplitude pico a pico do reflexo- H do estímulo condicionado e do estímulo não-condicionado. A média do estímulo condicionado será expresso em forma percentual em relação à média do estímulo não-condicionado para cada intervalo (Hr2/Hr1 x 100) ([PANIZZA et al., 1995](#_ENREF_7)).

## Plano de análise dos dados

Todas as variáveis contínuas coletadas terão sua normalidade testa por meio do teste Shapiro-Wilk. Uma vez confirmada a normalidade dos dados referentes à avaliação da excitabilidade cortical e medular, será aplicada a ANOVA de medidas repetidas (6 x 4), sendo considerados os seguintes fatores: tipo de estimulação (ETCCm anódica, ETCCm catódica, ETCCm sham, EMTr-20Hz, ETMr-1Hz e EMT sham) e tempo (antes, imediatamente após, 30 e 60 minutos após cada sessão). O teste t pareado será utilizado como post hoc. Para os casos em que a normalidade não for confirmada, será aplicado o teste de Kruskal-Wallis e o Wilcoxon. Todos os dados serão analizados por meio do software Statistical Package for Social Sciences (versão 20.0, SPSS Inc, Chicago IL, USA). Um p valor < 0.05 será considerado como significativo para todas as análises.

1. **Referências**

BENITO, J.; KUMRU, H.; MURILLO, N.; COSTA, U.; MEDINA, J.; TORMOS, J. M.; PASCUAL-LEONE, A.; VIDAL, J. Motor and gait improvement in patients with incomplete spinal cord injury induced by high-frequency repetitive transcranial magnetic stimulation. **Top Spinal Cord Inj Rehabil**, 18, 2, 106-112, Spring, 2012.

COGIAMANIAN, F.; VERGARI, M.; SCHIAFFI, E.; MARCEGLIA, S.; ARDOLINO, G.; BARBIERI, S.; PRIORI, A. Transcutaneous spinal cord direct current stimulation inhibits the lower limb nociceptive flexion reflex in human beings. **Pain**, 152, 2, 370-375, Feb, 2011.

FILIPOVIC, S. R.; ROTHWELL, J. C.; BHATIA, K. Slow (1 Hz) repetitive transcranial magnetic stimulation (rTMS) induces a sustained change in cortical excitability in patients with Parkinson's disease. **Clin Neurophysiol**, 121, 7, 1129-1137, Jul, 2010.

GARBER, C. E.; BLISSMER, B.; DESCHENES, M. R.; FRANKLIN, B.; LAMONTE, M. J.; LEE, I.-M.; NIEMAN, D. C.; SWAIN, D. P. American College of Sports Medicine position stand. Quantity and quality of exercise for developing and maintaining cardiorespiratory, musculoskeletal, and neuromotor fitness in apparently healthy adults: guidance for prescribing exercise. **Medicine and science in sports and exercise**, 43, 7, 1334-1359, 2011.

HERMENS, H. J.; FRERIKS, B.; DISSELHORST-KLUG, C.; RAU, G. Development of recommendations for SEMG sensors and sensor placement procedures. **J Electromyogr Kinesiol**, 10, 5, 361-374, Oct, 2000.

LIU, H.; AU-YEUNG, S. S. Reliability of transcranial magnetic stimulation induced corticomotor excitability measurements for a hand muscle in healthy and chronic stroke subjects. **J Neurol Sci**, 341, 1-2, 105-109, Jun 15, 2014.

PANIZZA, M.; BALBI, P.; RUSSO, G.; NILSSON, J. H-reflex recovery curve and reciprocal inhibition of H-reflex of the upper limbs in patients with spasticity secondary to stroke. **American journal of physical medicine & rehabilitation**, 74, 5, 357-363, 1995.

PELLICCIARI, M. C.; BRIGNANI, D.; MINIUSSI, C. Excitability modulation of the motor system induced by transcranial direct current stimulation: a multimodal approach. **Neuroimage**, 83, 569-580, Dec, 2013.

WINKLER, T.; HERING, P.; STRAUBE, A. Spinal DC stimulation in humans modulates post-activation depression of the H-reflex depending on current polarity. **Clin Neurophysiol**, 121, 6, 957-961, Jun, 2010.


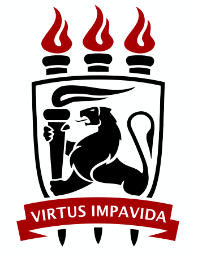
**UNIVERSIDADE FEDERAL DE PERNAMBUCO**

**CENTRO DE CIÊNCIAS DA SAÚDE**

**PROGRAMA DE PÓS-GRADUAÇÃO EM NEUROPSIQUIATRIA E CIÊNCIAS DO COPORTAMENTO**

**Main project:** Non-invasive central nervous system stimulation associated with locomotor training with body weight support in the recovery of spinal cord injured patients.

**Subproject:** Effect of different types of non-invasive central nervous system stimulation on pain perception, cortical and spinal cord excitability in healthy individuals.

**Researcher:** Plínio Luna de Albuquerque

**Supervisor:** Dr^a^. Kátia Karina do Monte Silva

**CAAE:** 41231715.6.0000.5208

# 1. Study design

It is a crossover, double-blind, sham-controlled, pseudorandomized, and counterbalanced study.

# 2. Location of the study

The study will be carried out at the Laboratory of Applied Neuroscience (LANA) of the Federal University of Pernambuco (UFPE).

# 3. Study period

The study duration is expected from March 2015 to November 2016.

# 4. Study population

The study population will be composed of healthy volunteers.

# 5. Sample

# 5.1 Sampling

Volunteers will be recruited through electronic media at the Federal University of Pernambuco. After screening the individuals will be selected and invited to participate in the study.

**5.2 Sample size**

The GPower software, version 3.1.3 for Windows (Franz Faul, Universität Kiel, Germany) was used to calculate the sample size. The calculation was performed according to the means and standard deviations of the withdrawal reflex amplitude and Hoffmann reflex of the study of Cogiamanian et al. (2011). The level of significance (α) adopted for the sample calculation was 0.05 and the power (β) adopted was 0.80.

# 5. 3 Eligibility Criteria

The sample will be composed of individuals who meet the following criteria: (i) healthy individuals (self-reported); (ii) male or female; (iii) age between 18 to 50 years. Will be not included in the study individuals who have a metal implant or any other metallic device in the body; pregnancy; history of seizures and epilepsy; presence of pacemaker and neurological disorders. Additionally, will be not included volunteers who are taking anxiolytic medications, antidepressants or any other medication that alters the level of cortical excitability.

# 6. Criteria for discontinuation

Individuals who report some discomfort during procedures or who no longer wish to participate in the study at any time during data collection will be discontinued from the study.

# 7. Experimental procedures

# 7.1 Randomization and allocation concealment

The randomization will be generated by a computer program, which will determine the order of sessions with the stimulation techniques. If necessary, part of the randomization may be altered (pseudorandomized), in order to allow the counterbalanced sequence among the volunteers.

The order of the sessions of each subject will be coded and transferred to a series of opaque envelopes numbered to the volunteers selected for the study. The envelopes will be sent to the researcher 2, who is responsible for the stage of application of the stimulation, leaving the researcher 1 blind as to the technique of stimulation that the volunteer will be submitted to.

# 7.2 Baseline assessment

Initially, the researcher will explain the study’s aim, after this, all subjects will be orientated about the free participation and they will invited to sign the Consent Form. Only after the authorization, the subjects will be invited to respond to a semi-structured questionnaire with epidemiological data (age, gender, marital status, education level, etc.) and, they will be asked about their usual caffeine-containing foods (amount, frequency and type of food) during the last 24 hours, medication in use, and amount of sleep. For women, the date of the last menstrual period will be identified.

# 7.3 Central nervous system non-invasive stimulation

Each subject will be underwent six experimental sessions containing a baseline assessment of cortical and spinal excitability followed by a randomly assigned non-invasive stimulation session, tsDCS (anodal, cathodal or sham) or TMS (20 Hz, 1Hz or Sham), tested in a double-blind condition. The stimulation setting and electrode positioning will be made by a different physician not involved in enrollment to guarantee the blinding of the assessors. Additionally, the neurophysiological acquisition and data analysis will be made by blinding assessors regarding the kind of non-invasive stimulation. An external researcher will be responsible for the allocation process and the final allocation will keeped in sealed envelopes.

All evaluations and interventions will be performed at the same shift of the day. Additionally, the motivation and fatigue levels will be evaluated before the start of each session to guarantee the same basal condition. After the each session of rTMS or tsDCS all subjects will underwent to 20 minutes of treadmill training with moderate intensity following immediate (T0), 30 minutes (T30) and 60 min (T60) after stimulation re-assessment. To avoid any inﬂuence by previous stimulations, one week washout between sessions will be adopted.

The trans-spinal direct current stimulation will be delivered by an eletric stimulator (Neuroconn®/Germany) connected to a pair of electrodes (7 cm x 5 cm) of saline-soaked synthetic sponge positioned between eleventh and twelveth thoracic vertebras (active electrode) and on right shoulder (reference electrode). The electrode will placed on the spinal cord will determine the type of the tsDCS. The stimulation will be administered for 1,200s, fade in and fade off 10s with an intensity of 2,5 mA and, a current density of 0,071 mA/cm². The sham tsDCS will follow the same montage of anodal stimulation but after 30 s the stimulator will be turned off. The sham stimulation will provide the same initial sensation of active stimulation but will not induce neurophysiological changes. A questionnaire about adverse effects will be apply for all individuals after each session of active or sham tsDCS.

Repetitive Transcranial Magnetic Stimulation will be delivered by an eight coil attached to a magnetic stimulator (Rapid Magstin®). All individuals will seated in a comfortable chair with headrest and armrests. The rest motor threshold (RMT) of the first dorsal interosseous (FID) muscle will be determined on the left motor cortex before starting the stimulation. The high frequency repetitive stimulation protocol will be composed by a total of 1800 pulses, delivered by two trains with two seconds of duration at a frequency of 20Hz (40 pulses/train) and 28 seconds of interval. The low frequency repetitive stimulation protocol (1Hz rTMS) will be composed by a total of 1500 pulses. For both conditions, a repetitive stimulation will be applied over the FID hotspot with an intensity of 90% of motor threshold. The control group will be composed by the sham rTMS. For this condition, a coil disconnected from the stimulator unit will be held over the scalp while a second coil, connected with the stimulator, will be positioned behind the patient’s head, without touching the scalp. The sham rTMS will not able to induce effects in the brain tissue, but will exposed the subjects to the same acoustic stimulation.

# 7.4 Treadmill exercise protocol

A 20 minutes treadmill walk (Gait training- Biodex) with moderate intensity (64% to 76% of maximum heart rate) will be performed after each session of rTMS or tsDCS. The maximum heart rate (HRmax) will be calculated using the following formula: HRmax = 220 – Age. In order to guarantee similar exercise intensity for all individuals, at every three minutes the subject perceived exertion and heart rate will be assessed by Borg scale and monitored by cardiac polar (Polar RS800, Polar Electro- Kempele, Finland). Additionally, treadmill inclination and speed will be controlled to avoid fatigue or light training.

# 7.5 Evaluation of the cortical excitability

The Neuro-MS will be used to evaluate the corticospinal excitability. The evaluation of the electrical brain activity will be performed through transcranial magnetic stimulation by single pulse (TMS-p) and by paired pulse (TMS-pp). The analysis of motor cortex excitability will be performed through: (i) determination of resting motor threshold and (ii) amplitude of motor evoked potential (MEP).

All volunteers will be instructed to sit on a chair in a comfortable position. Initially, TMS-p stimuli will be administered over the motor cortex to determine the area related to the ​​cortical representation of the first dorsal interosseous muscle (FID). To record the electromyographic activity, two electrodes, with a diameter of 10 mm will be placed on the belly of the target muscle, and one reference electrode will be positioned at the interphalangeal joint of the thumb. All the care for the electromyographic signal acquisition will be taken according to the criteria of the Surface Electromyography for the Non-invasive Assessment of Muscles (SENIAM) (HERMENS et al., 2000).

To locate the representation of the FID muscle, the coil with a 45 degree angulation will be positioned at a distance of 20% from the Cz point (according to the international 10/20 marking system), towards the contralateral tragus to the muscle to be evaluated (PELLICCIARI; BRIGNANI & MINIUSSI, 2013; LIU & AU-YEUNG, 2014). During the evaluation, an angled coil of eight will be used to avoid calibration bias. The following measures will be carried out:

1. Resting motor threshold (RMT) – The RMT will considered as the lowest single-pulse TMS intensity required to produce a motor evoked potential amplitude larger than 50mV (confirmed by surface electromyography). The Motor Threshold Assessment Tool - MTAT 2.0 (http://www.clinicalresearcher.org/software.htm) was used to assess the RMT.
2. Motor-evoked potential (MEP) - To determine the MEP, the intensity of the magnetic stimulator will be adjusted to 130% of the RMT. With the individuals positioned comfortably, twenty pulses will be delivered and the electromyographic response will be captured. The mean peak to peak amplitude of the potentials will be considered to determine the average of the cortical excitability (FILIPOVIC; ROTHWELL & BHATIA, 2010).

# 7.6 Evaluation of the Spinal Cord excitability

The spinal cord excitability will be evaluated through three different measures: (i) the Hoffmann reflex; (ii) Homosynaptic reflex and (iii) Nociceptive flexion reflex.

(i) Hoffmann reflex (Hr): To assess Hr and homosynaptic depression, all individuals will be positioned comfortably on lying position. The lower limbs will be placed with knee flexed 30º and ankle at 90º. The Hoffmann reflex (Hr) will be elicited by eletrical stimulator (Neuromep-8, Russia). Electric stimuli will be delivered to the tibial nerve at popliteal fossa and the electrophysiological records will be made by self-adhesive Ag–AgCl electrodes placed on the medial portion of the right soleus (1.0 cm diameter) with 2 cm interelectrode distance. The ground electrode will be positioned over gastrocnemius midline 10 cm from to the recorded electrodes.

To obtain the maximal Hoffman reflex amplitude (Hrmax) and maximal M-wave amplitude (Mmax), we will delivered rectangular pulses with 1.0 ms, every 12 seconds. To build a recruitment cure, a current intensity will be increased in steps of 1 mA. All the records will be collected at a bandwidth of 5 and 10000 Hz and, a sample rate of 20000 Hz. To minimize physiological artefacts, the impedance will kept below 3 kΩ.

(ii) Nociceptive flexion reflex (NFR): To evoke the NFR, five consecutive electrocutaneous stimuli will be delivered over the sural nerve through Neuro-Mep bar electrodes (a bipolar stimulation electrode with cathode placed on superior extremity) applied behind the right lateral malleolus. The stimulation trial will consist of five rectangular pulses with 1ms duration and intensity equivalent to 120% of NFR threshold. To record NFR activity, a electromyographic pair of surface electrodes (Ag/AgCl, inter-electrode distance of 2 cm) will be positioned on the belly of ipsilateral brevis head of the biceps femoris muscle of the right leg and a reference (common ground electrode) on lateral epicondyle of femur. To avoid the stimulus predictability the stimulation will be delivered randomly (5 to 20s) without previous warning. In cases where the volunteer reported a rating of 10, the threshold assessment will be discontinued.

1. Homosynaptic depression: To assess the homosynaptic depression we will performe a H-reflex recovery curve. For this, we will plott the Hr recovery curve by delivering pairs of stimuli following the same setting described above with the same intensity and duration used to elicit the maximal Hoffmann reflex aplitude. Stimuli will be delivered at different interestimulus interval 40, 50, 70, 75, 100, 150, 200, 250, 300, 350, 400, 450, 500, 600, 700, 800 and 900 ms, and 1, 2, 3, 4 and 5 seconds at frequency no faster than 12 seconds. The mean of peak-to-peak Hr amplitude of the unconditioned and conditioned H-reflex will be calculated. Conditioned mean will be expressed as a percentage of the unconditioned mean for every delay (Hr2/Hr1 x 100).

# Statistical analysis

All continuous data will have their normality tested through the Shapiro-Wilk test. After confirming the normality of the cortical and spinal excitability data, the repeated measures ANOVA (6 x 4) will be applied considering the following factors: stimulation (anodal tsDCS, cathodal tsDCS, sham tsDCS, 20Hz rTMS, 1 Hz rTMS and sham rTMS) and time (before, immediately after, 30 and 60 minutes after each session). The paired t-test will be used as post hoc. For those cases where normality will be not confirmed, the Kruskal-Wallis and the Wilcoxon test will be applied. All data will be analyzed using the Statistical Package for Social Sciences (version 20.0, SPSS Inc, Chicago IL, USA). A P value of < 0.05 will be considered significant for all statistical analysis.

1. **References**

BENITO, J.; KUMRU, H.; MURILLO, N.; COSTA, U.; MEDINA, J.; TORMOS, J. M.; PASCUAL-LEONE, A.; VIDAL, J. Motor and gait improvement in patients with incomplete spinal cord injury induced by high-frequency repetitive transcranial magnetic stimulation. **Top Spinal Cord Inj Rehabil**, 18, 2, 106-112, Spring, 2012.

COGIAMANIAN, F.; VERGARI, M.; SCHIAFFI, E.; MARCEGLIA, S.; ARDOLINO, G.; BARBIERI, S.; PRIORI, A. Transcutaneous spinal cord direct current stimulation inhibits the lower limb nociceptive flexion reflex in human beings. **Pain**, 152, 2, 370-375, Feb, 2011.

FILIPOVIC, S. R.; ROTHWELL, J. C.; BHATIA, K. Slow (1 Hz) repetitive transcranial magnetic stimulation (rTMS) induces a sustained change in cortical excitability in patients with Parkinson's disease. **Clin Neurophysiol**, 121, 7, 1129-1137, Jul, 2010.

GARBER, C. E.; BLISSMER, B.; DESCHENES, M. R.; FRANKLIN, B.; LAMONTE, M. J.; LEE, I.-M.; NIEMAN, D. C.; SWAIN, D. P. American College of Sports Medicine position stand. Quantity and quality of exercise for developing and maintaining cardiorespiratory, musculoskeletal, and neuromotor fitness in apparently healthy adults: guidance for prescribing exercise. **Medicine and science in sports and exercise**, 43, 7, 1334-1359, 2011.

HERMENS, H. J.; FRERIKS, B.; DISSELHORST-KLUG, C.; RAU, G. Development of recommendations for SEMG sensors and sensor placement procedures. **J Electromyogr Kinesiol**, 10, 5, 361-374, Oct, 2000.

LIU, H.; AU-YEUNG, S. S. Reliability of transcranial magnetic stimulation induced corticomotor excitability measurements for a hand muscle in healthy and chronic stroke subjects. **J Neurol Sci**, 341, 1-2, 105-109, Jun 15, 2014.

PANIZZA, M.; BALBI, P.; RUSSO, G.; NILSSON, J. H-reflex recovery curve and reciprocal inhibition of H-reflex of the upper limbs in patients with spasticity secondary to stroke. **American journal of physical medicine & rehabilitation**, 74, 5, 357-363, 1995.

PELLICCIARI, M. C.; BRIGNANI, D.; MINIUSSI, C. Excitability modulation of the motor system induced by transcranial direct current stimulation: a multimodal approach. **Neuroimage**, 83, 569-580, Dec, 2013.

WINKLER, T.; HERING, P.; STRAUBE, A. Spinal DC stimulation in humans modulates post-activation depression of the H-reflex depending on current polarity. **Clin Neurophysiol**, 121, 6, 957-961, Jun, 2010.
