# Supplementary material for: Effects of repetitive transcranial magnetic stimulation and trans-spinal direct current stimulation associated with treadmill exercise in spinal cord and cortical excitability of healthy subjects: A triple-blind, randomized and sham-controlled study
Source: PLoS One. 2018 Mar 29;13(3):e0195276. doi: 10.1371/journal.pone.0195276 (PMC5875883; doi:10.1371/journal.pone.0195276)
Supplement: S3 Text — (PDF) [file pone.0195276.s004.pdf]

PARECER CONSUBSTANCIADO DO CEP

DADOS DA EMENDA

Título da Pesquisa: ESTIMULAÇÃO NÃO INVASIVA DO SISTEMA NERVOSO CENTRAL ASSOCIADA AO TREINO LOCOMOTOR COM SUPORTE DE PESO CORPORAL NA RECUPERAÇÃO DE PACIENTES LESADOS MEDULARES

Pesquisador: PLÍNIO LUNA DE ALBUQUERQUE

Área Temática:

Versão: 3

CAAE: 41231715.6.0000.5208

Instituição Proponente: CENTRO DE CIÊNCIAS DA SAÚDE

Patrocinador Principal: Financiamento Próprio

DADOS DO PARECER

Número do Parecer: 1.571.394

Apresentação do Projeto:

Trata-se de emenda ao projeto de pesquisa na qual o pesquisador solicita: (1) acrescentar três novos pesquisadores na equipe de pesquisa; (2) incluir uma nova fase (um estudo crossover) antes do ensaio clínico; (3) incluir uma frequência de estimulação não invasiva do sistema nervoso central (EMTr 1 Hz); (4) reduzir a quantidade de sessões na terceira etapa do projeto de 15 para 12 sessões e, (5) adequar o TCLE para contemplar as três fases do estudo.

Tais modificações foram justificadas a seguir.

(1) Os três novos pesquisadores serão necessários para cumprimento de todas as etapas do projeto no tempo previsto no cronograma.

(2) Ao longo da execução da primeira fase do estudo o pesquisador percebeu a necessidade de testar em indivíduos com lesão medular a estimulação não invasiva do sistema nervoso central que apresentou melhores resultados quanto à modulação da atividade cortical e medular de indivíduos saudáveis. A inclusão de uma fase com indivíduos com lesão medular servirá como estudo piloto para a terceira etapa do projeto, esta última, com maior duração. As informações referentes a metodologia da fase II no projeto está descritas nas páginas 15, 16, 23-27, 33, 35 e 37.

(3) Os resultados parciais da primeira fase do estudo indicaram que apenas a EMTr de 20 Hz foi capaz de modular a excitabilidade cortical e medular simultaneamente em indivíduos saudáveis. No entanto não se sabe se a estimulação de baixa frequência (EMTr de 1 Hz) também seria capaz de promover os mesmos resultados, uma vez que esta estimulação não estava presente no projeto inicial. Dessa maneira, a inclusão da EMT 1Hz se faz necessário antes do início do ensaio clínico com pacientes com lesão medular. Os parâmetros para a EMT 1Hz estão descritos na página 27.

(4) a redução da quantidade de sessões na terceira etapa do projeto de 15 para 12 sessões justifica-se para garantir a viabilidade do projeto no cronograma proposto e com os recursos disponíveis, mediante a inclusão de mais uma etapa no estudo. Estudos recentes que utilizaram treino de marcha com suporte de peso corporal, como proposto por este estudo, evidenciam que protocolos com doze sessões já beneficiam a melhora da marcha em indivíduos com lesão medular incompleta. A modificação da quantidade de sessões encontra-se descrita na página 30 do projeto detalhado.

(5) As alterações no TCLE foram propostas para contemplar a nova formatação do projeto e fornecer informações claras aos indivíduos incluídos na pesquisa. O TCLE modificado encontra-se no arquivo anexado à Plataforma Brasil separadamente (TCLE) e no final do projeto detalhado nas páginas 49 e 50,

Objetivo da Pesquisa:

Identificar a técnica de estimulação não invasiva do Sistema Nervoso Central (SNC) que quando associada ao treino de marcha com suporte de peso corporal é mais eficiente em modificar a excitabilidade cortical, medular e percepção de dor de indivíduos saudáveis.

Avaliação dos Riscos e Benefícios:

O estudo oferece pouco risco à saúde dos participantes, uma vez que as avaliações que serão realizadas já são bem estabelecidas na literatura científica e serão realizadas sob a supervisão de pesquisadores experientes na área. Pode-se considerar um risco que alguns métodos de avaliação dos voluntários possam deixá-los constrangidos ou desconfortáveis, porém todas as informações obtidas mediante a coleta serão mantidas sob sigilo, respeitando assim a privacidade dos indivíduos.

Dentre os principais benefícios oferecidos pelo estudo estão à avaliação neurofisiológica da

atividade elétrica do córtex cerebral e do segmento medular. A partir dos resultados destas avaliações pode-se compreender a relação entre o estado em que se encontram os circuitos neuronais no córtex e na medula e os sinais clínicos apresentados por cada paciente participante da pesquisa. Outro benefício encontra-se nas sessões de treinamento de marcha na esteira com suporte de peso corporal que será oferecido a todos os pacientes incluídos no estudo. O treinamento com suporte de peso corporal já possui evidências na recuperação sensório motora de pacientes com lesão medular incompleta, no entanto, devido ao seu alto custo aquisição apenas é encontrado em grandes centros de reabilitação. Durante o desenvolvimento do estudo e ao termino das coletas os pacientes receberão orientações sobre os cuidados com a pele e prevenção de úlceras de pressão, exercícios para evitar a instalação de contraturas musculares e deformidades articulares. Após as orientações os exercícios serão realizados pelo voluntário sob a supervisão dos pesquisadores a fim de orientá-los a respeito da forma correta de realizá-los.

Comentários e Considerações sobre a Pesquisa:

O presente projeto de pesquisa propõe-se através de um ensaio clínico investigar a inovação terapêutica da associação do treino de marcha com suporte de peso com técnicas de estimulação não invasiva do SNC na tentativa de promover uma recuperação mais efetiva e rápida dos pacientes. Em adição, será realizado um estudo prévio em indivíduos saudáveis, de modo a identificar, qual técnica de estimulação altera em maior magnitude a excitabilidade cortical e medular para depois investigar sua eficácia em aperfeiçoar o processo de reabilitação de pessoas com lesão medular.

Considerações sobre os Termos de apresentação obrigatória:

Conteúdo avaliado anteriormente pelo CEP tendo sido considerado aprovado.

Recomendações:

Sem recomendações.

Conclusões ou Pendências e Lista de Inadequações:

Emenda adequadamente apresentada.

Considerações Finais a critério do CEP:

A emenda foi avaliada e APROVADA pelo colegiado do CEP.

Este parecer foi elaborado baseado nos documentos abaixo relacionados:

Tipo Documento	Arquivo	Postagem	Autor	Situação

Informações Básicas do Projeto	PB_INFORMAÇÕES_BÁSICAS_695103 E1.pdf	09/04/2016 22:25:38		Aceito
Outros	Justificativa_emenda.doc	09/04/2016 22:23:28	PLÍNIO LUNA DE ALBUQUERQUE	Aceito
Projeto Detalhado / Brochura Investigador	PROJETO_DETALHADO.doc	09/04/2016 22:21:30	PLÍNIO LUNA DE ALBUQUERQUE	Aceito
TCLE / Termos de Assentimento / Justificativa de Ausência	TCLE.doc	09/04/2016 21:28:24	PLÍNIO LUNA DE ALBUQUERQUE	Aceito
Outros	Rodrigo_Lattes.pdf	09/04/2016 21:23:53	PLÍNIO LUNA DE ALBUQUERQUE	Aceito
Outros	Luis_Lattes.pdf	09/04/2016 21:21:55	PLÍNIO LUNA DE ALBUQUERQUE	Aceito
Outros	Thyciane_Lattes.pdf	09/04/2016 21:21:07	PLÍNIO LUNA DE ALBUQUERQUE	Aceito
Folha de Rosto	Plinio.jpg	02/02/2015 10:27:00		Aceito
Outros	Carta de anuência.jpg	02/02/2015 10:07:50		Aceito
Outros	Lattes_Adriana_Baltar.pdf	01/02/2015 18:35:31		Aceito
Outros	Lattes_Livia_Shi.pdf	01/02/2015 18:34:52		Aceito
Outros	Lattes_Plínio Luna.pdf	01/02/2015 18:34:34		Aceito
Outros	Lattes_Katia.pdf	01/02/2015 18:33:42		Aceito

Situação do Parecer:
Aprovado

Necessita Apreciação da CONEP:
Não

RECIFE, 02 de Junho de 2016

Assinado por:
LUCIANO TAVARES MONTENEGRO
(Coordenador)
